# Supplementary material for: Mechanical ordering of pigment crystallites in oil binder: can electron paramagnetic resonance reveal the gesture of an artist?
Source: Magn Reson (Gott). 2022 Nov 22;3(2):211–20. doi: 10.5194/mr-3-211-2022 (PMC10539773; doi:10.5194/mr-3-211-2022)
Supplement: Demonstration of Eq. (7); numerical calculation of the coefficients pl from experimental EPR spectra; Matlab script for the calculation of the orientation probability density; experimental and calculated EPR spectra. The supplement related to this article is available online at: https://doi.org/10.5 [file mr-3-211-supplement.pdf]

Supplement of Magn. Reson., 3, 211–220, 2022
https://doi.org/10.5194/mr-3-211-2022-supplement
© Author(s) 2022. CC BY 4.0 License.

Supplement of

Mechanical ordering of pigment crystallites in oil binder: can electron paramagnetic resonance reveal the gesture of an artist?

Elise Garel et al.

Correspondence to: Laurent Binet (laurent.binet@chimieparistech.psl.eu)

The copyright of individual parts of the supplement might differ from the article licence.

1. Demonstration of Equation (7)

The Wigner matrix elements $D_{00}^{(l)}(\Omega)$ in Eq. 6 can be expressed as (Eq. 12 in Hentschel et al., J. Chem. Phys. 68, 56-66, https://doi.org/10.1063/1.435473, 1978):

$$D_{00}^{(l)}(\Omega) = \sum_{n=-l}^{l} D_{0n}^{(l)}(\Omega') D_{0n}^{*(l)}(\Omega_0) \qquad (S1)$$

Then Eq. 4 becomes:

$$S(B,\Omega_0) = \sum_{l=0}^{\infty} p_l \left\{ \sum_{n=-l}^{l} \left[D_{0n}^{*(l)}(\Omega_0) \int_{\Omega'} D_{0n}^{(l)}(\Omega')\omega(\Omega') f\big(B - B_r(\Omega')\big) d\Omega' \right] \right\} \qquad (S2)$$

where the integration over Ω is replaced by an integration over Ω' since a set of orientations (Ω, Ω_0) determines an orientation Ω' in a bi-univocal way. In the above equation:

$$\int_{\Omega'} D_{0n}^{(l)}(\Omega')\omega(\Omega') f\big(B - B_r(\Omega')\big) d\Omega' = \int_{\varphi=0}^{2\pi} d\varphi \int_{\theta=0}^{\pi} f\big(B - B_r(\theta)\big) \sin\theta \, d\theta \int_{\psi=0}^{2\pi} \omega(\varphi,\theta,\psi) D_{0n}^{(l)}(\varphi,\theta,\psi) d\psi \qquad (S3)$$

For weakly anisotropic g-factor as is the case here, $\omega(\varphi,\theta,\psi)$ is almost constant and will be dropped afterwards as it then appears as an irrelevant scaling factor.

From the property:

$$\int_{\psi=0}^{2\pi} D_{0n}^{(l)}(\varphi,\theta,\psi) d\psi = \begin{cases} 0 \text{ if } n \neq 0 \\ 2\pi D_{00}^{(l)}(0,\theta,0) \text{ if } n = 0 \end{cases} \qquad (S4)$$

we get:

$$S(B,\Omega_0) = (2\pi)^2 \sum_{l=0}^{\infty} p_l D_{00}^{*(l)}(\Omega_0) \int_{\theta=0}^{\pi} D_{00}^{(l)}(0,\theta,0) f\big(B - B_r(\theta)\big) \sin\theta \, d\theta \qquad (S5)$$

and since $D_{00}^{*(l)}(\Omega_0) = P_l(\cos\beta_0)$ and $D_{00}^{(l)}(0,\theta,0) = P_l(\cos\theta)$, we finally get Eq. 7:

$$S(B,\Omega_0) = S(B,\beta_0) = \sum_{l=0}^{\infty} p_l P_l(\cos\beta_0) \int_{\theta=0}^{\pi} f\big(B - B_r(\theta)\big) P_l(\cos\theta) \sin\theta \, d\theta \qquad (S6)$$

after dropping the irrelevant $(2\pi)^2$ factor.

2. Numerical calculation of the coefficients p_l from experimental EPR spectra

For practical implementation, the discrete summation over *l* in Eq. S6 must be truncated to a finite order l_{max}. In addition, EPR spectra are recorded for a finite discrete set of *m* field values $(B_i)_{i=1,\cdots,m}$. For a specific orientation $\beta_{0,k}$ $(k = 1, \cdots, n)$ in a set of *n* different orientations of the sample in the laboratory frame, we then define a matrix $\mathbf{G}(\beta_{0,k})$ with elements:

$$G_{ij}(\beta_{0,k}) = P_{2(j-1)}(\cos\beta_{0,k}) \int_{\theta=0}^{\pi} f\big(B_i - B_r(\theta)\big) P_{2(j-1)}(\cos\theta) d\cos\theta \qquad (S7)$$

with $j = \frac{l}{2} + 1$, which runs from 1 to $N = \frac{l_{max}}{2} + 1$ by step of 1 when l runs from 0 to l_{max} by step of 2, since l is even. In practice, the integral in Eq. S7 is replaced by a discrete summation and a Voigt line shape is used for $f\big(B_i - B_r(\theta)\big)$. The theoretical EPR spectrum at orientation $\beta_{0,k}$ is then expressed as an *m*-line column vector:

$$\boldsymbol{S}_k^{th} = \mathbf{G}(\beta_{0,k}) \begin{pmatrix} p_0 \\ \vdots \\ p_{l_{max}} \end{pmatrix} \tag{S8}$$

The best estimates of the unknown coefficients $\left(p_0, \cdots, p_{l_{max}}\right)$ are obtained by minimizing:

$$L^2 = \sum_{k=1}^{n} \left\| \boldsymbol{S}_k^{exp} - \boldsymbol{S}_k^{th} \right\|^2 \tag{S9}$$

which represents the squared distance between all theoretical spectra $\boldsymbol{S}_k^{th}$ and experimental spectra $\boldsymbol{S}_k^{exp}$, with respect to the variations of the coefficients p_l. The best estimates are then given by:

$$\begin{pmatrix} p_0 \\ \vdots \\ p_{l_{max}} \end{pmatrix} = \left(\sum_{k=1}^{n} \mathbf{G}(\beta_{0,k})^T \mathbf{G}(\beta_{0,k}) \right)^{-1} \left(\sum_{k=1}^{n} \mathbf{G}(\beta_{0,k})^T \boldsymbol{S}_k^{exp} \right) \tag{S10}$$

where the upper script *T* denotes the transposed matrix.

3. Matlab script for the calculation of the orientation probability density

```
clear;

[B_exp_angle,spc_exp,Params]=eprload('Experimental spectrum file name');

% B_exp_angle: cell containing the experimental magnetic field values and
the rotation angles
% spc_exp: experimental EPR spectra at different orientations
% Params : cell containing the EPR acquisition settings
% eprload : Function in Easyspin allowing the loading of EPR spectra in
Bruker format


B_exp_angle{1,1}=B_exp_angle{1,1}/10; % G to mT conversion of magnetic
fields

size_Bexp=size(B_exp_angle{1,1});
index_point_B250=find(B_exp_angle{1,1}>=250); % indices of all points such
as field >= 250 mT
index_point_B400=find(B_exp_angle{1,1}>=400);%indices of all points such as
field >= 400 mT

% Restriction of the experimental spectra to 250 mT < field <400 mT
Bmax=B_exp_angle{1,1}(index_point_B400(1));
Bmin=B_exp_angle{1,1}(index_point_B250(1));
B_sweep= Bmax-Bmin; % magnetic field interval width

length_spc_exp=size(spc_exp);
for i=1:length_spc_exp(2)
    spc_exp(:,i)=spc_exp(:,i)-spc_exp(index_point_B400(1),i); %base line
set to 0
end

  %PLOT THE SUPERPOSED SPECTRA SET
  %{
figure;
hold on
for j=1:2:19
    plot(B_exp_angle{1,1},spc_exp(:,j))
end
hold off
  %}


 Sys.S=1/2; % Electron spin of Cu2+
 Sys.g=[2.055 2.34]; % [g_perpendicular g_parallel] g-factors for Cu2+


Param.mwFreq = Params.MWFQ/10^9;% Experimental microwave frequency
Param.Range = [Bmin Bmax];% Magnetic field range

[phi,theta,weight]= sphgrid('Dinfh',100); % Set of orientations of the
magnetic field in the molecular frame for a magnetic system with axial
symmetry

Param.CrystalOrientation = transpose([phi; theta; weight]);
```

```
[pos, amp, wid, trans]= resfields(Sys, Param); % resfields : Easyspin
function to compute the resonance fields (pos) and transition probabilities
(amp)

% Calculation of the EPR spectra

nb_trans= size(pos); % number of calculated EPR transitions
nb_orientations=size(theta); % number of field orientations in the
molecular frame
beta0=B_exp_angle{1,2}*pi/180; % conversion of angles in radian
max_spectre=19; % number of spectra at different sample orientations

max_order=16; % highest order of the Legendre polynomials in the expansion
of the probability density P(beta)

A_l=[];
for l=0:2:max_order
    sum=0;
    for i=1:nb_trans(1)
        for k=1:nb_orientations(2)
            w_i_thetak=amp(i,k);
            P_l_thetak= legendreP(l,cos(theta(k)));

            B_res_i_k=pos(i,k);
            if isnan(B_res_i_k)==0
                fwhmGL=[0
8.6+sqrt(6.1^2*cos(theta(k))^2+3.4^2*sin(theta(k))^2)]; % width at half
maximum for gaussian and lorentzian component of the EPR line shape
                f_B_Bres_i_k=
voigtian(B_exp_angle{1,1}(index_point_B250(1):index_point_B400(1)),
B_res_i_k, fwhmGL,1);
                sum= sum + P_l_thetak* w_i_thetak * f_B_Bres_i_k *
weight(1,k);
            end
        end
    end
    A_l=[A_l, transpose(2*pi*sum)];
end

G={};
sum_GS=0;
sum_GG=0;
for num_spectrum=1:max_spectre
    G_beta = [];
    for l=0:2:max_order
        P_l_beta=legendreP(l,cos(beta0(num_spectrum)));
        G_beta= [G_beta,P_l_beta*A_l(:,l/2+1)];
    end
    G{end+1}=G_beta;
    sum_GS = sum_GS + transpose(G_beta) *
spc_exp(index_point_B250:index_point_B400,num_spectrum) ;
    sum_GG= sum_GG +transpose(G_beta)*G_beta;
end

p_matrix= inv(sum_GG)*sum_GS;

    % Plot of experimental and calculated spectra
```

```
for num_spectrum_reconstitute=1:1:max_spectre
    spc_th= G{1,num_spectrum_reconstitute}* p_matrix; % Calculates the
theoretical EPR spectrum

    figure;

plot(B_exp_angle{1,1}(index_point_B250(1):index_point_B400(1)),transpose(sp
c_th),B_exp_angle{1,1}(index_point_B250(1):index_point_B400(1)),spc_exp(ind
ex_point_B250:index_point_B400,num_spectrum_reconstitute));

end


% Plot of the probability density

betas=linspace(0,pi,480);
distrib_beta_i=[];
distrib_deconv=[];
for beta=betas
    legendres=[];
    for l=0:2:max_order
        legendres = [legendres, legendreP(l,cos(beta))];
    end
    proba_beta_i = legendres*p_matrix;
    distrib_beta_i=[distrib_beta_i, proba_beta_i];
end

distrib_beta_i_norm=distrib_beta_i/trapz(betas,4*pi*pi*distrib_beta_i.*sin(
betas)); % normalisation of the probability density

p_matrix=p_matrix/trapz(betas,4*pi*pi*distrib_beta_i.*sin(betas));%
normalisation of the p_l coefficients


figure;
polarplot([betas,betas+pi], [distrib_beta_i_norm,distrib_beta_i_norm])%
plot in polar coordinate the orientation probability density
hold on
polarplot([betas,betas+pi], 1/(8*pi*pi)*ones(2*size(betas)))% plots the
isotropic probability density
```

3. Experimental and calculated EPR spectra

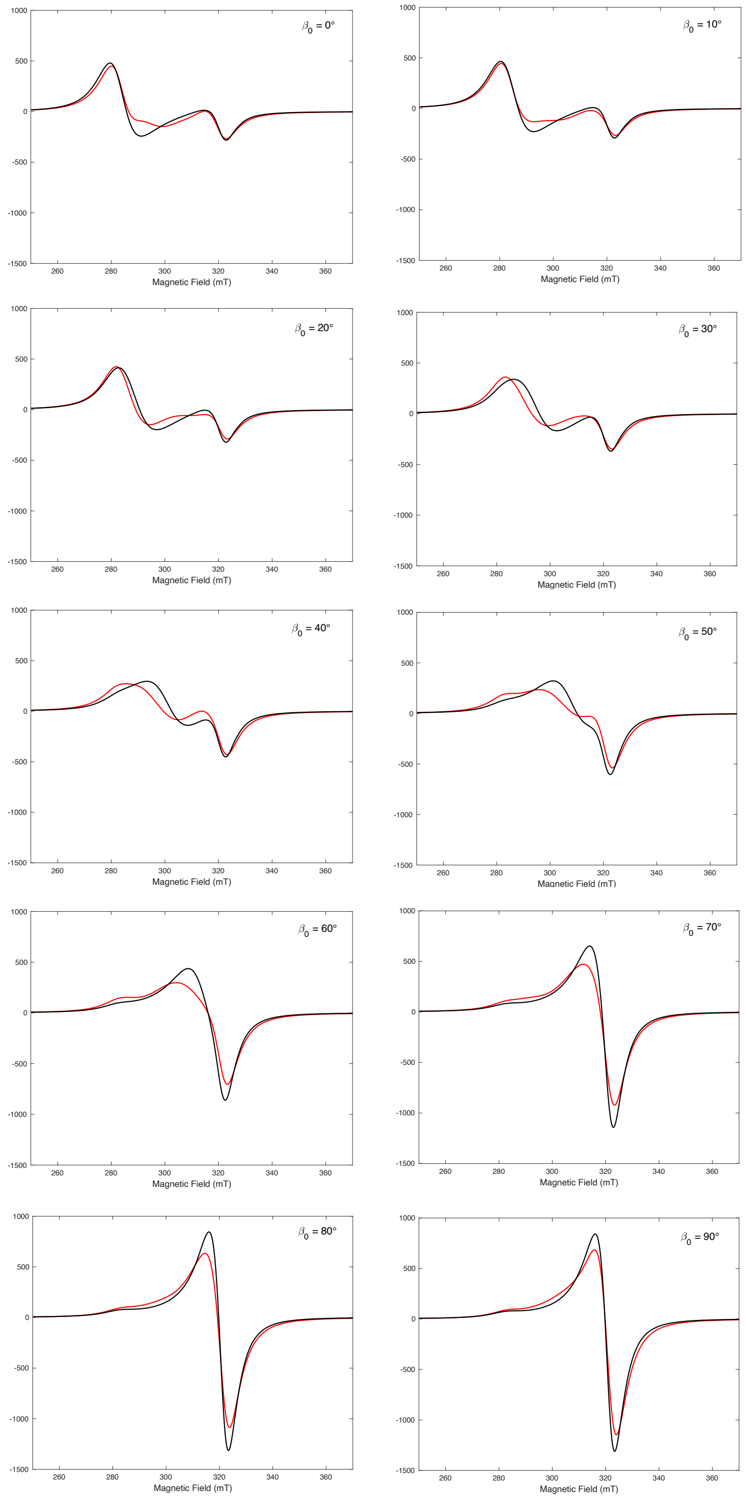

Figure S1. Experimental (black) and calculated (red) EPR spectra of microcrystals of cuprorivaite magnetically oriented in fluid oil for different orientations of the measuring field with respect to the sample Z-axis, upon rotation about the Y-axis. The rotation axis is Y_0 and the sample was set with $Y \parallel Y_0$.

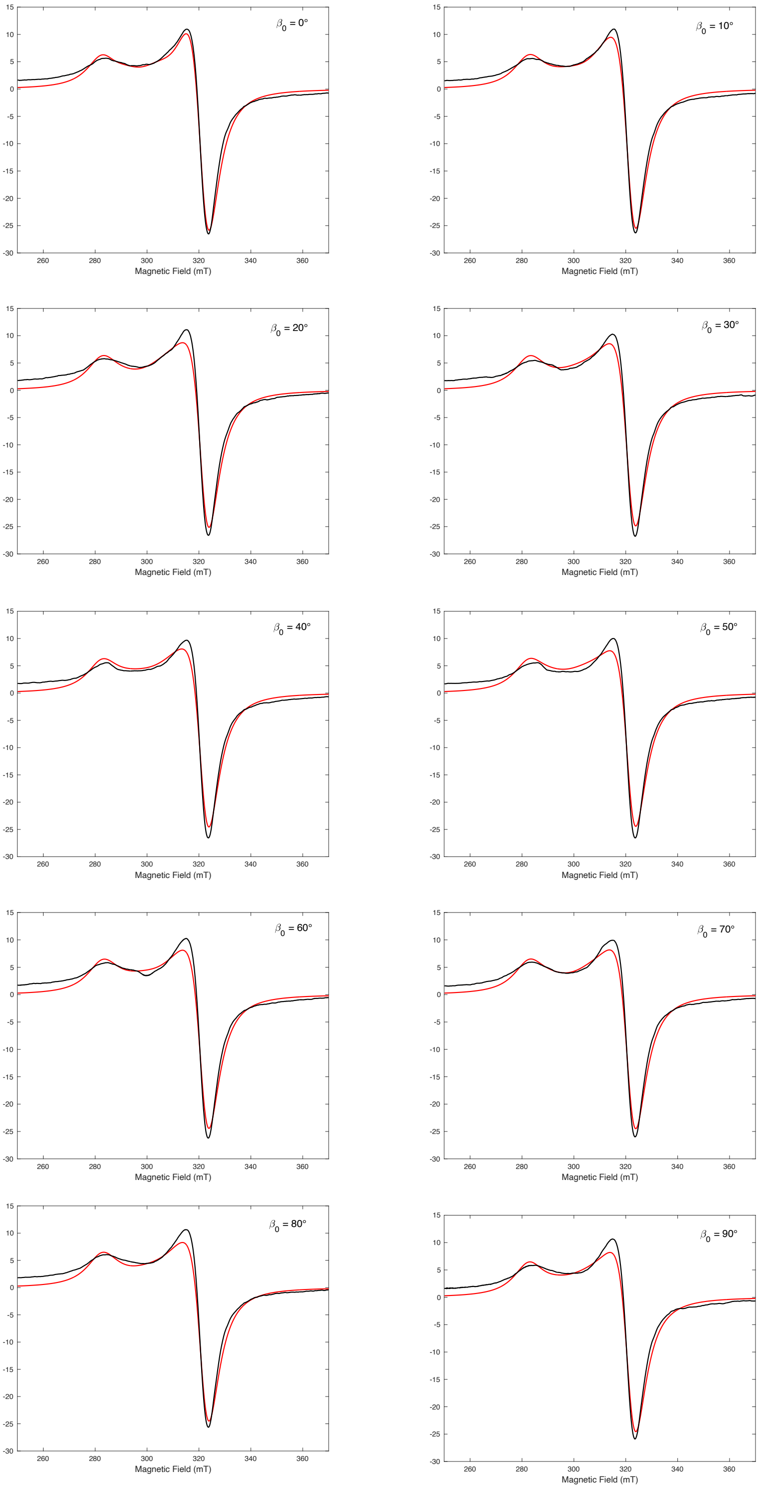

Figure S2. Experimental (black) and calculated (red) EPR spectra of microcrystals of cuprorivaite in a dried oil film deposited with an applicator for different orientations of the measuring field with respect to the sample *X*-axis, upon rotation about the *Z*-axis. The rotation axis is Y_0 and the sample was set with $Z \parallel Y_0$.

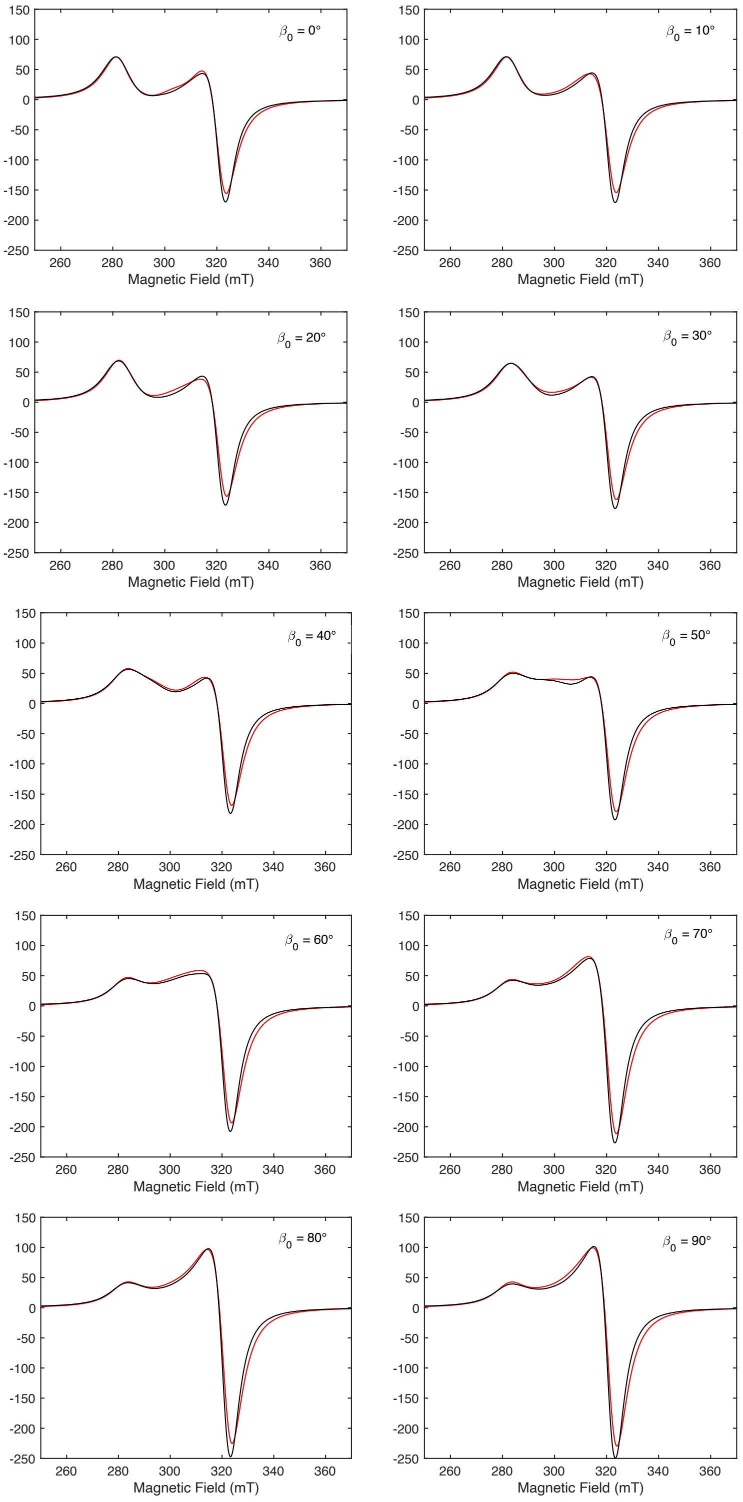

Figure S3. Experimental (black) and calculated (red) EPR spectra of microcrystals of cuprorivaite in a dried oil film deposited with an applicator for different orientations of the measuring field with respect to the sample Z-axis, upon rotation about the Y-axis. The rotation axis is Y_0 and the sample was set with $Y \parallel Y_0$.

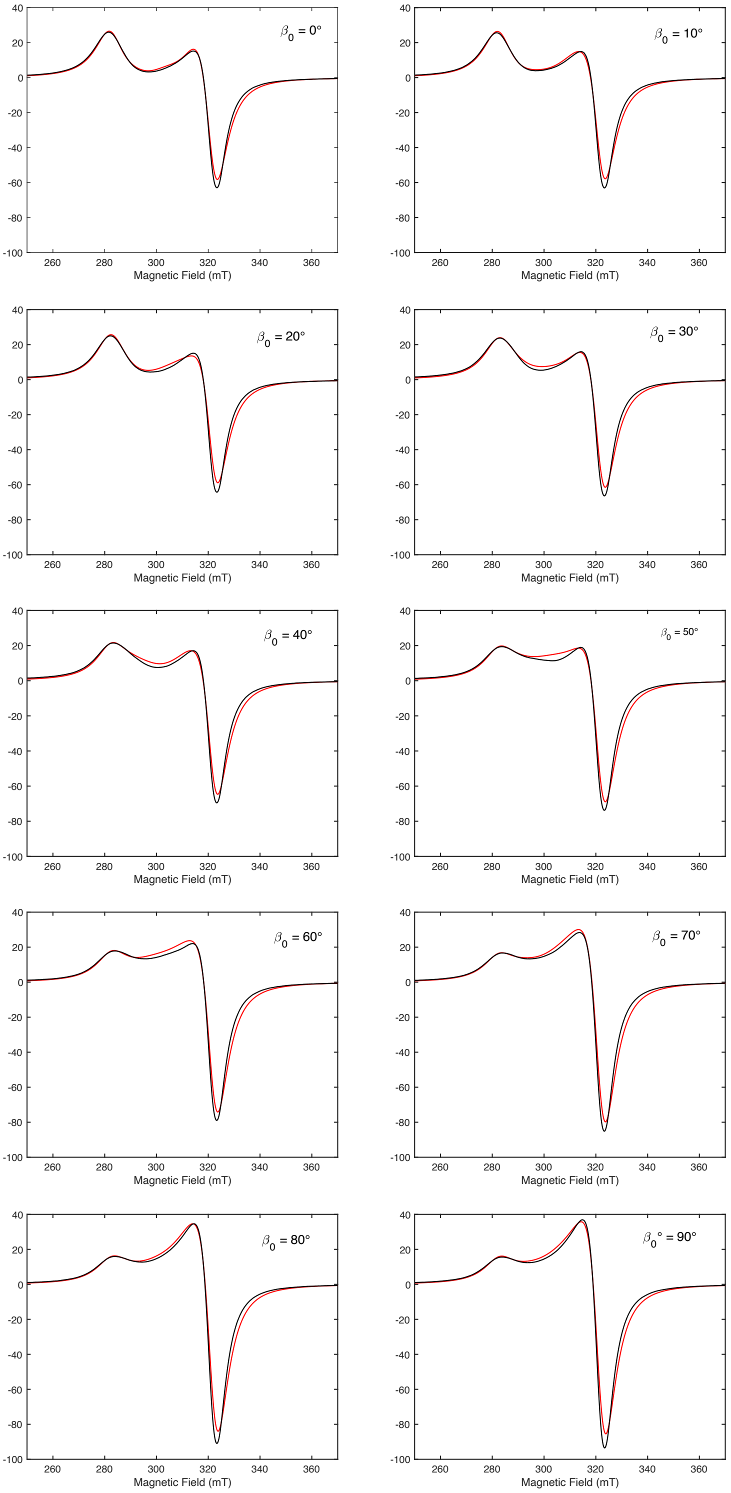

Figure S4. Experimental (black) and calculated (red) EPR spectra of microcrystals of cuprorivaite in a dried oil film deposited with paintbrush for different orientations of the measuring field with respect to the sample *Z*-axis, upon rotation about the *Y*-axis. The rotation axis is Y_0 and the sample was set with $Y \parallel Y_0$.

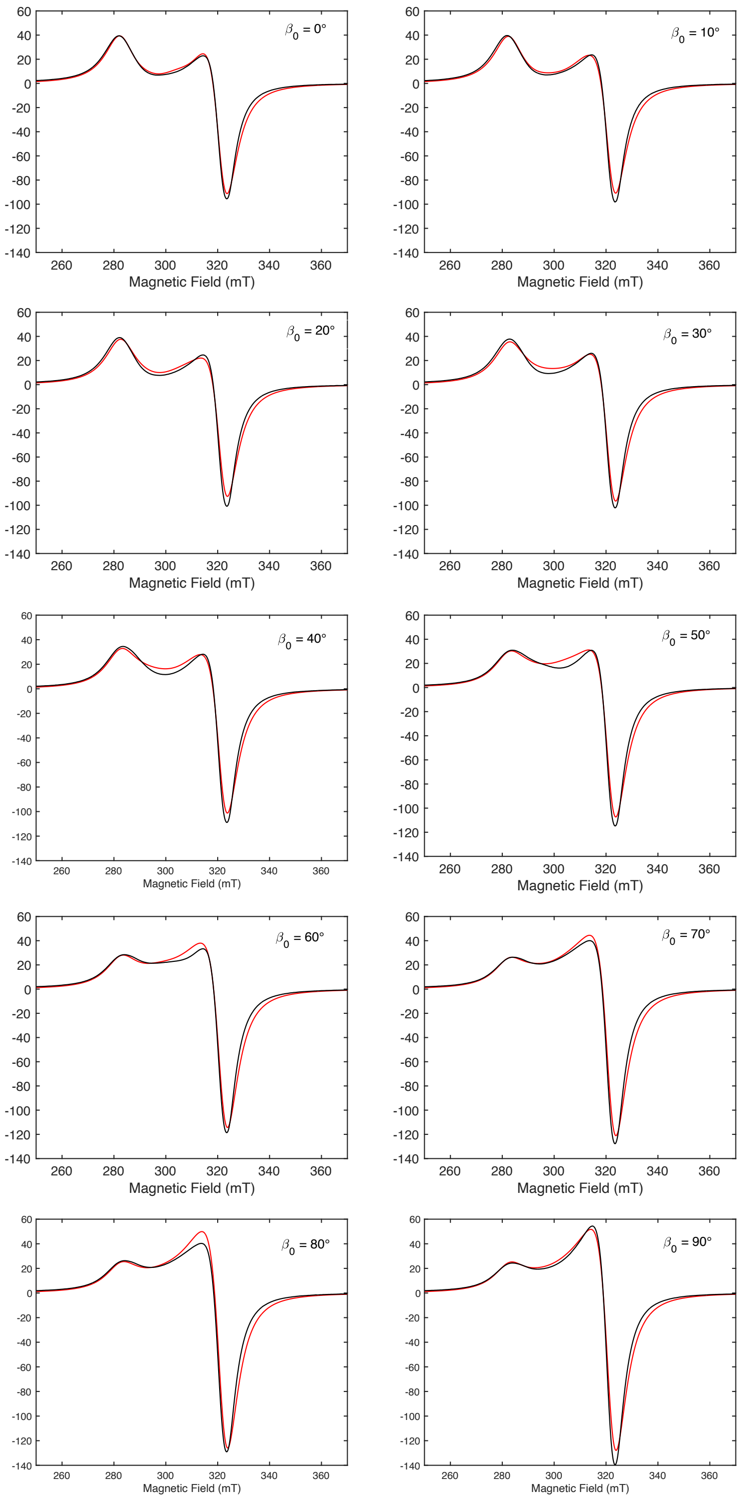

Figure S5. Experimental (black) and calculated (red) EPR spectra of microcrystals of cuprorivaite in a dried oil deposited as a droplet for different orientations of the measuring field with respect to the sample *Z*-axis, upon rotation about the *Y*-axis. The rotation axis is Y_0 and the sample was set with $Y \parallel Y_0$.

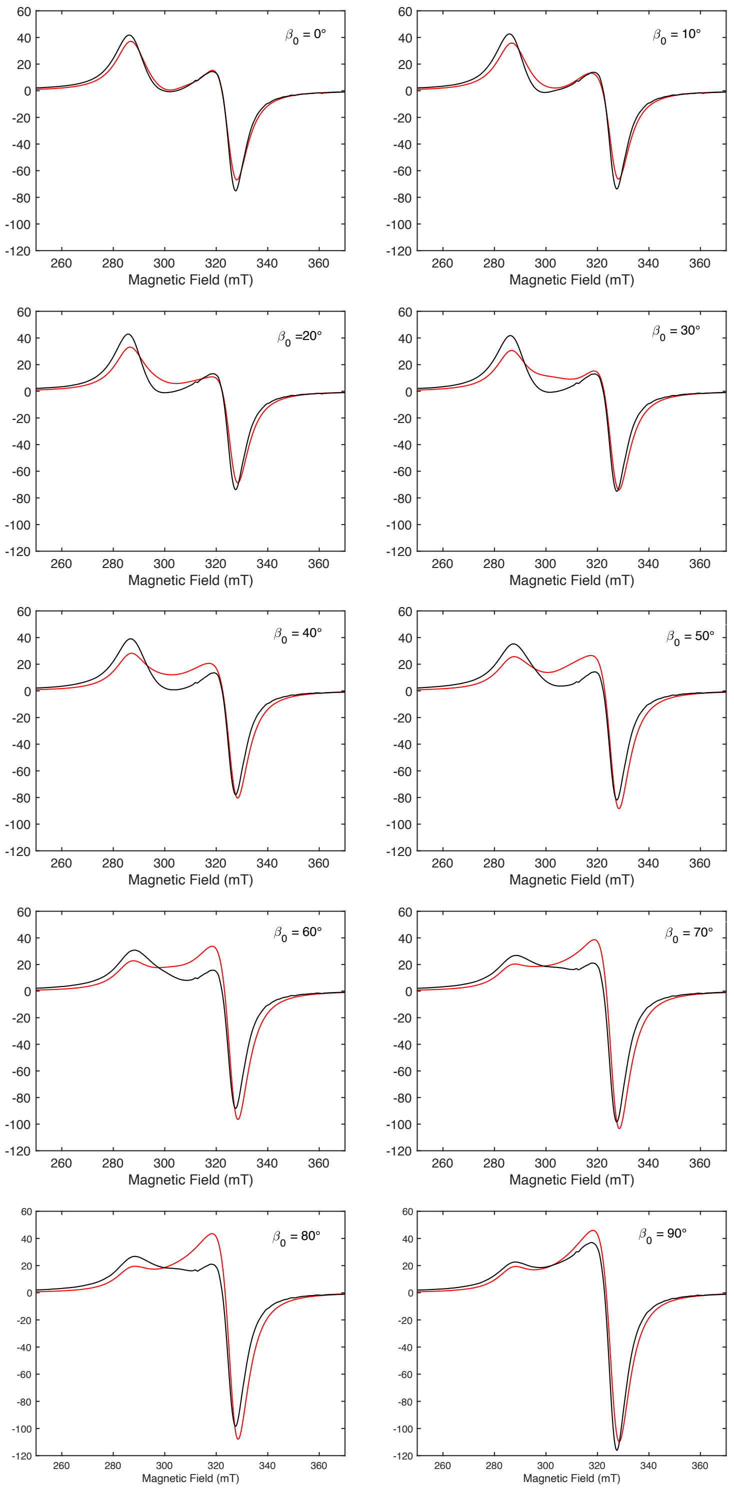

Figure S6. Experimental (black) and calculated (red) EPR spectra of microcrystals of cuprorivaite in a dried oil deposited by dabbing for different orientations of the measuring field with respect to the sample Z-axis, upon rotation about the Y-axis. The rotation axis is Y_0 and the sample was set with $Y \parallel Y_0$.
